# Body Sodium Overload Modulates the Firing Rate and Fos Immunoreactivity of Serotonergic Cells of Dorsal Raphe Nucleus

**DOI:** 10.1371/journal.pone.0074689

**Published:** 2013-09-20

**Authors:** Andrea Godino, Soledad Pitra, Hugo F. Carrer, Laura Vivas

**Affiliations:** 1 Instituto de Investigación Médica Mercedes y Martín Ferreyra (INIMEC-CONICET-Universidad Nacional de Córdoba), Córdoba, Argentina; University of California, Los Angeles, United States of America

## Abstract

In order to determine whether serotonergic (5HT) dorsal raphe nucleus (DRN) cells are involved in body sodium status regulation, the effect of a s.c. infusion of either 2 M or 0.15 M NaCl on 5HT DRN neuron firing was studied using single unit extracellular recordings. In separate groups of 2 M and 0.15 M NaCl-infused rats, water intake, oxytocin (OT) plasma concentration, urine and plasma sodium and protein concentrations were also measured. Also, to determine the involvement of particular brain nuclei and neurochemical systems in body sodium overload (SO), animals from both groups were perfused for brain immunohistochemical detection of Fos, Fos-OT and Fos-5HT expression. SO produced a significant increase in serotonergic DRN neuron firing rate compared to baseline and 0.15 M NaCl-infused rats. As expected, 2 M NaCl s.c. infusion also induced a significant increase of water intake, diuresis and natriuresis, plasma sodium concentration and osmolality, even though plasma volume did not increase as indicated by changes in plasma protein concentration. The distribution of neurons along the forebrain and brainstem expressing Fos after SO showed the participation of the lamina terminalis, extended amygdala, supraoptic and paraventricular hypothalamic nuclei in the neural network that controls osmoregulatory responses. Both Fos-OT immunoreactive and plasma OT concentration increased after s.c. hypertonic sodium infusion. Finally, matching the *“in vivo”* electrophysiological study, SO doubled the number of Fos-5HT immunolabeled cells within the DRN. In summary, the results characterize the behavioral, renal and endocrine responses after body sodium overload without volume expansion and specify the cerebral nuclei that participate at different CNS levels in the control of these responses. The electrophysiological approach also allows us to determine in an “*in vivo*" model that DRN 5HT neurons increase their firing frequency during an increase in systemic sodium concentration and osmolality, possibly to modulate sodium and water intake/excretion and avoid extracellular volume expansion.

## Introduction

Under normal volemia conditions, increased natremia or plasma osmolality involves compensatory responses such as water intake, sodium appetite inhibition, plasma release of oxytocin (OT) and vasopressin (AVP), and consequently renal sodium excretion and water retention [1]. The central circuit involved in the control of these behavioral and physiological responses induced by hypernatremia has been studied, analyzing the expression of brain immediate early genes [2]–[4]. Enhanced *c-fos* expression as shown by increased Fos immunoreactivity was identified within the magnocellular groups of OT and AVP hypothalamic paraventricular and supraoptic nuclei (PVN and SON), and matching the observed increase of both peptides in plasma [4], [5]. The increased natremia and osmolality is centrally detected by the circumventricular organs of the lamina terminalis (CVOs of LT), which have osmo- and sodium-sensitive cells and have shown increased Fos immunoreactivity (Fos-ir) after different paradigms of body salt loading [4]–[6]. At brainstem level, this stimulus involved the activity of the lateral parabrachial nucleus (LPBN), locus coeruleus (LC), ventrolateral medulla (VL), nucleus of the solitary tract (NTS) and area postrema (AP). Together, all these areas work in coordination to mediate the autonomic, endocrine and behavioral responses inherent in osmoregulation [4], [7], [8].

Our previous results involved the serotonergic neurons of the dorsal raphe nucleus (DRN) in the regulation of fluid and electrolyte balance after particular hydroelectrolyte disturbances [9]–[14]. These studies analyzed the double immunoreactive cells for Fos and serotonin along the raphe system, before and after sodium intake induced by sodium depletion and after extracellular volume expansion (EVE). The data indicate that body sodium status modulates the activity of serotonergic DRN neurons, since Fos-5HT-ir decreased after sodium depletion and increased in animals in positive sodium balance or in process of reestablishing sodium balance. Serotonergic cells of the DRN were also activated after body sodium status was reestablished, independently of the concentration of NaCl consumed, suggesting that this system is involved in the inhibition of sodium appetite under conditions of satiety [11]. Significant increased activity was also observed in serotonergic DRN cells after EVE [10]. Moreover, our connectional studies using a retrograde marker, Fluorogold, in combination with Fos have demonstrated an important functional projection from the DRN to the LPBN and the LT, which are involved in different models of fluid and sodium balance regulation [12]–[14].

In agreement with the above evidence, other authors have demonstrated that DRN lesion and 5HT antagonist (Methysergide) injection into the LPBN increased sodium intake induced by sodium depletion and decreased sodium and potassium renal excretion and endocrine responses (OT and AVP) after EVE. Similarly, the 5HT agonist (5HT2A/2C, 2,5-dimethoxy-4-iodoamphetamine hydrobromide), injected into the LPBN, increased the renal and endocrine response after EVE and inhibited sodium intake induced by different experimental models [15]–[20].

Together these results suggest that body sodium levels modulate the activity of serotonergic DRN cells. However, their specific participation in a model of systemic hypernatremia/hyperosmolarity without blood volume expansion, directly recording “in vivo” DRN cell electrical activity during sodium overload, has not yet been studied. For this purpose, anesthetized animals were used, subjected to a single unit extracellular recording of serotonergic DRN cells and receiving a s.c. infusion of either 2 M or 0.15 M NaCl (0.6 ml/100 g b w) solution for one minute. The distribution of neurons along the forebrain and brainstem that express Fos, the double immunolabeling of Fos-5HT, and Fos-OT were also identified under this sodium overload model, and behavioral, renal and endocrine responses were characterized.

Our electrophysiological study demonstrates that the firing rate of putative 5HT DRN neurons increases after body sodium overload, matching the pattern of Fos-5HT double immunostaining. This suggests that the serotonergic system within the DRN participates in the brain circuit that controls this homeostatic response. Oxytocinergic neural activity and OT plasma concentration also increased after sodium overload without EVE, confirming their involvement in this physiological condition. The central circuit activated after body sodium overload involves specific nuclei along the brainstem, lamina terminalis, hypothalamic and central extended amygdala areas.

## Materials and Methods

The experiments used adult, Wistar-derived male rats, born and reared in the breeding colony at the Instituto Ferreyra (INIMEC, Córdoba, Argentina). Animals weighing 250–300 g were housed singly in metabolic cages. Room lights were on for 12 h/day, and temperature was controlled at 23°C.

### Ethical Statement

All experimental procedures were approved and carried out in accordance with the guidelines of the Ethical Committee of the Instituto Ferreyra for the use and care of laboratory animals. The protocol designed to minimize animal suffering was approved by this Committee (Permit Number: PIP 2009/2011, 11220080102561).

### Sodium Overload (So)

After 4 h of food and water deprivation, each adult rat was weighed, injected subcutaneously (s.c.) on the back with xilocaine (in an effort to reduce non-specific treatment-associated pain and stress), and 5 minutes later with hypertonic saline (2 M NaCl) delivered through a 26-gauge needle (0.6 ml/100 g body weight, for 1 minute) using an infusion pump (B. Braun, Germany), and then returned to its metabolic cage. Control rats were injected similarly but with isotonic saline (0.15 M NaCl). Individual rats from each group were used for urine and plasma measurements, OT radioimmunoassay or brain immunohistochemical detection of Fos, Fos-OT and Fos-5HT expression as described below.

Another group of animals was used for the electrophysiological study, and were anesthetized and subjected to SO (as previously described) during the extracellular recording.

### Experiment 1: Analysis of the Firing Frequency of Serotonergic DRN Neurons during Sodium Overload

The rats were anesthetized with urethane 50% (0.3 ml/100 g b w) and then were catheterized in the jugular vein. Body temperature was maintained at 36–37°C, with a feedback-controlled heating pad. The animals were mounted onto a stereotaxic frame, their skulls exposed, and a hole was drilled above the DRN into which an electrode was lowered (7.8 mm posterior to bregma, 1.0 mm lateral to midline suture, 5.5–7.0 mm below the dura mater) [21] by means of a hydraulic microdrive. The saggital sinus was ligated and displaced to avoid puncturing this sinus during the descent of the electrode. Single unit DRN discharge was recorded with glass micropipettes (1 µm tip diameter recording electrode, World Precision Instruments, Inc., item number 1B120F-4) filled with 2 M NaCl solution containing 2% Pontamine Sky Blue. The firing rate was obtained from cells which displayed a signal: noise ratio 2∶1 or more. DRN serotonergic neurons were tentatively identified on the basis of characteristic firing patterns and pharmacological characterization for an “*in vivo”* or “*in vitro”* spontaneously active neurons described previously by Baraban & Aghajanian (1980), Kirby et al., (2003) and others [22]–[26]. These features includes: a slow (0.5–2.5 Hz), regular firing rate and biphasic action potentials of 1.0–3.0 ms duration, while pharmacological phenotype characterization was performed by 5HT1A stimulation induced by iv injection of fluoxetine (a serotonin reuptake inhibitor which causes a decrease in the firing frequency of serotonergic neurons mainly by 5HT1A autoreceptors stimulation). Thus, we only recorded the spontaneously active neurons which have a slow and regular firing rate that are expected to be inhibited by 5HT1A autoreceptors stimulation [24]. Electrode potentials, which had been previously passed through a high-impedance amplifier, were passed through a window discriminator and screened on an audio amplifier. Integrated histograms generated by the analogy output of the window discriminator were analyzed on-line with a computer.

Only one neuron was recorded per animal in order to analyze the response to 2 M NaCl or 0.15 M NaCl injection. The baseline activity of each neuron was recorded for 3–5 min before any treatment, and changes in neuronal firing were observed for 15 min after s.c. saline infusions (2 M or 0.15 M NaCl). The mean discharge rate was determined over 1 min intervals. Pharmacological phenotype characterization was then performed by iv injection of fluoxetine. The recording site was marked by injecting Pontamine sky blue dye using a pneumatic pressure pump (Medical Systems Corp. NY MS-2).

For the qualitative analysis, the following criteria were used to characterize the neuronal response to hypertonic or isotonic infusions:

Excitatory response: when firing frequency increases 0.2 Hz or more after infusion in comparison to baseline activity.
Inhibitory response: when firing frequency decreases 0.2 Hz or more after infusion in relation to baseline.
Neutral response: the absolute changes in firing frequency after infusion do not exceed 0.2 Hz in relation to baseline activity.

For quantitative analysis, the registered putative 5HT cells were analyzed using a two-way ANOVA mixed with repeated measures (treatment as main factor and time as repeated measures) was used. Post-hoc comparisons were made with the least significant difference test.

### Experiment 2: Physiological Characterization of the Response to Sodium Overload

#### Water intake induced by SO

Food- and water-deprived animals were s.c. saline infused (2 M NaCl or 0.15 M NaCl), returned to metabolic cages without food and provided immediate access to water. Cumulative intake was measured in calibrated burettes at 0, 30, 60, 120, and 180 min. Drinking latencies were recorded immediately after saline infusion. A one-way repeated measures ANOVA was used for the analysis of water intake. Post-hoc comparisons were made with the least significant difference test.

#### Determination of plasma OT concentration

For the plasma OT concentration assay, different groups of animals were used from those used in the immunohistochemical and electrophysiological studies.

Animals were decapitated and bled before and 5, 10 and 15 minutes after SO. Trunk blood was collected in chilled plastic tubes containing heparin. Plasma OT level was measured by radioimmunoassay as described by Morris and Alexander (1989) [27]. For the assay, OT was extracted from 1 ml of plasma with acetone and petroleum ether. The percentage of recovery after extraction was 85%. The assay sensitivity and intra- and inter-assay coefficients of variation were 0.9 pg/ml, 7% and 12.6%. A two-way ANOVA was used for plasma oxytocin concentration analysis.

#### Electrolytes and protein assays

To analyze the plasma electrolyte concentration in the 2 M NaCl or 0.15 M NaCl groups, animals were decapitated and bled before and 5, 10 and 15 minutes after SO. Samples were centrifuged and 1 ml of plasma was extracted and stored at −20°C. Urinary samples were taken after the intake test (180 min. after s.c. infusion) and immediately centrifuged, and 1 ml was extracted and stored at −20°C. Electrolyte concentrations of these samples were analyzed by flame photometry (Hitachi 911, automatic analyzer). Plasma volume was inferred from the plasma protein concentration measured according to Lowry [28]. Plasma and urine osmolality were analyzed by vapor pressure osmometer (VAPRO 5520). A two-way ANOVA (treatment and time factors) was used for the analysis of plasma electrolytes, protein concentration and osmolality. Post-hoc comparisons were made with the least significant difference test. A t-test was used for the urinary electrolytes and osmolality after treatments.

### Experiment 3: Brain Pattern of Fos, Fos-OT and Fos-5HT Immunoreactivity after SO

Animals were perfused 90 min after s.c. 2 M NaCl or 0.15 M NaCl infusion and their brains extracted for immunohistochemical detection of Fos, Fos-OT and Fos-5HT. For this purpose, the different groups of rats were anesthetized with thiopentone (100 mg/kg ip) and perfused transcardially with ∼100 ml normal saline followed by ∼400 ml of 4% paraformaldehyde in 0.1 M phosphate buffer (PB, pH 7.2). The 90 minutes interval after stimulation was chosen in order to be able to compare the distribution of sodium overload-induced c-fos in these animals with that observed and reported previously after induced-sodium consumption [9], [11], [29]. The brains were removed, fixed in the same solution overnight, and then stored at 4°C in PB containing 30% sucrose. Coronal sections were cut into two series of 40 µm using a freezing microtome and were placed in a mixture of 10% H_2_O_2_ and 10% methanol until oxygen bubbles ceased appearing. They were incubated in 10% normal horse serum (NHS) in PB for 1 h to block non-specific binding sites.

All the series of the free-floating sections from each brain were first processed for Fos immunoreactivity (Fos-ir), using an avidin-biotin-peroxidase procedure. The sections of the midbrain were then also stained for 5HT immunoreactivity (5HT-ir) and those from the hypothalamus for OT (OT-ir). The staining procedures followed the double-labeling procedures previously described [29], [9]. Briefly, free-floating sections were incubated overnight at room temperature in a rabbit anti-fos antibody (produced in rabbits against a synthetic 14-amino acid sequence, corresponding to residues 4–17 of human Fos) (Ab-5, Calbiochem), diluted 1∶10,000 in PB containing 2% NHS (Gibco, Auckland, NZ) and 0.3% Triton X-100 (Sigma Chemical Co., St. Louis, MO, USA). The sections were then washed with PB and incubated with biotin-labeled anti-rabbit immunoglobulin and avidin-biotin-peroxidase complex (Vector Laboratories Inc., Burlingame, CA USA, 1∶200 dilutions in 1% NHS-PB) for 1 h at room temperature. The peroxidase label was detected using diaminobenzidine hydrochloride (DAB, Sigma Chemical Co., St. Louis, MO, USA), intensified with 1% cobalt chloride and 1% nickel ammonium sulphate. This method produces a blue-black nuclear reaction product. The series of Fos-labeled sections, also processed for immunocytochemical localization of 5HT and OT, were incubated for 72 h at 4°C with their corresponding antibodies: polyclonal rabbit anti-5HT antibody (ImmunoStar Inc, WI, USA, dilution 1∶10,000) and polyclonal rabbit anti-OT antibody (Calbiochem, dilution: 1∶25,000). After incubation, the sections were rinsed and incubated with biotin-labeled anti-rabbit immunoglobulin and avidin-biotin-peroxidase complex for 1 h at room temperature. Cytoplasmic 5HT-ir and OT-ir were detected with unintensified DAB that produces a brown reaction product. Finally, the free-floating sections were mounted on gelatinized slides, air-dried overnight, dehydrated, cleared in xylene and placed under a coverslip with DePeX (Fluka, Buchs, Switzerland).

Fos-ir controls were conducted by placing sections in primary Fos antibody that had been preadsorbed with an excess of Fos peptide, or by processing sections without the primary antiserum. No Fos-ir neurons were observed following either of these control procedures.

### Cytoarchitectural and Quantitative Analysis

Brain nuclei exhibiting Fos-ir were identified and delimited according to the rat brain atlas of Paxinos and Watson [21]. The different PVN subnuclei were counted at three different levels of PVN, anterior, medial, posterior (distance from the bregma of the corresponding plates: −1.30 mm, −1.80 mm, −2.12 mm respectively). The distance from the bregma of the corresponding plates is indicated between brackets: accumbens core (AcbC, 1.00 mm), SON (−1.3 mm), central amygdaloid nucleus (CeA, −2.3 mm), bed nucleus of the stria terminalis, laterodorsal subdivision (BSTLD, −0.26 mm), subfornical organ (SFO, −0.92 mm), organum vasculosum of the lamina terminalis (OVLT, −0.20 mm), median preoptic nucleus (MnPO, −0.30 mm), thalamic anterodorsal (AD, −1.8 mm), median raphe nucleus (MnR) and DRN (−8.00 mm), LPBN (−9.3 mm), NTS (−13.24 mm) and AP (−13.68 mm).

Fos-ir nuclei were quantified using a computerized system that includes a Zeiss microscope equipped with a DC 200 Leica digital camera attached to a contrast enhancement device. Images were digitalized and analyzed using Scion Image PC, based on the NIH 1997 version. Fos-ir cells in each section were counted by setting a size range for cellular nuclei (in pixels) and a threshold level for staining intensity. Representative sections in each group were acquired at exactly the same level, with the aid of the Adobe Photoshop Image Analysis Program, version 5.5. The counting was done in four animals of each condition, and was repeated at least twice on each section analyzed, to ensure that the number of profiles obtained was similar. The investigator who conducted the counting of Fos-ir cells was blinded for the experimental groups. Immunohistochemical study was analyzed by Student t-test.

## Results

### Experiment 1: Effects of a Body Sodium Overload on Putative 5HT-DRN Neuronal Firing

**Figure 1:**
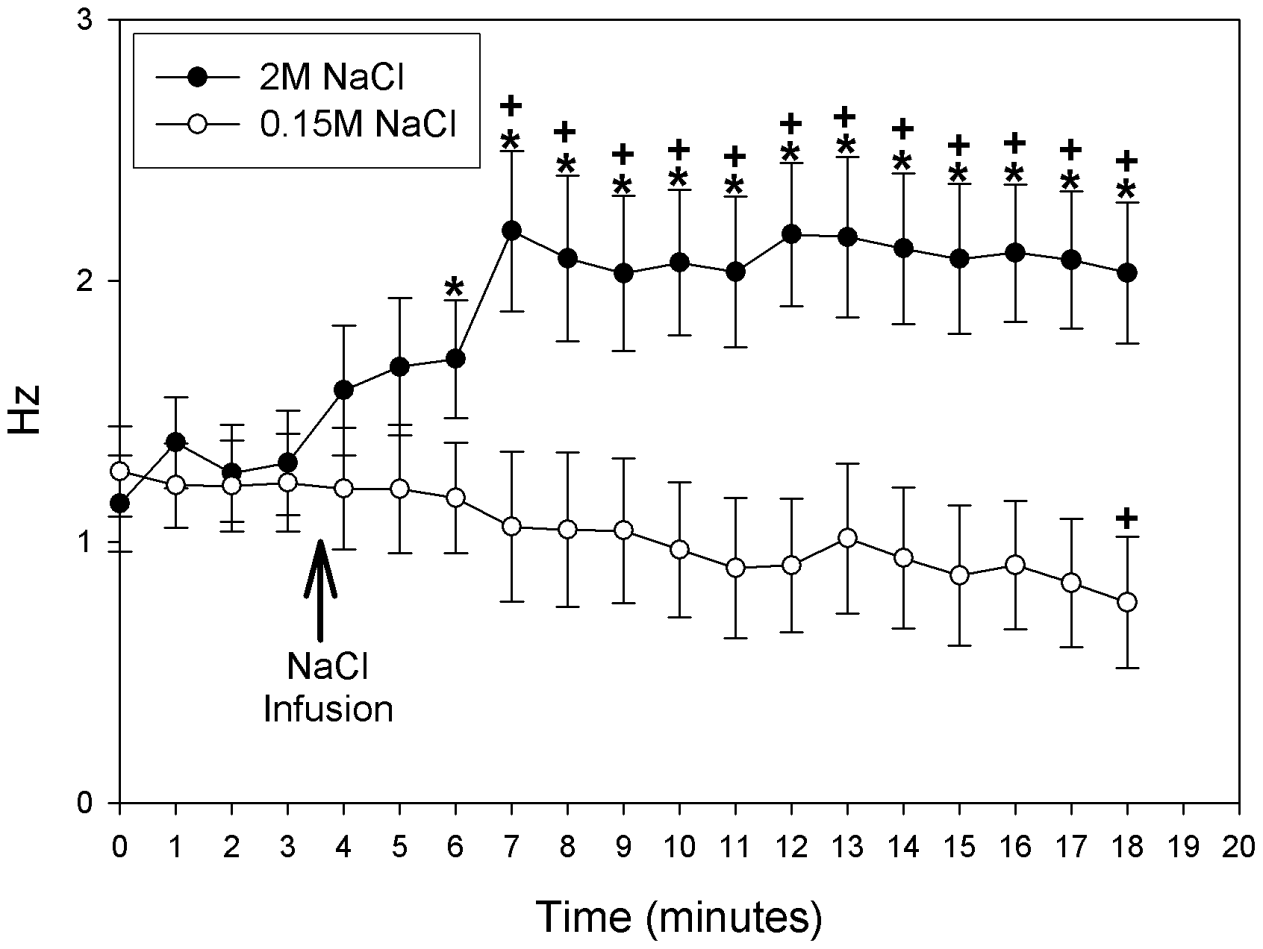
Effect of 2-DRN neurons. Values are means of 1 minutes intervals from 2(n = 14, black circles) or 0.15 M NaCl (n = 16, white circles) groups, before and after NaCl infusion indicated by the arrow. Values are means ± SE. *P<0.05 vs. 0.15 M NaCl group+P<0.05 vs. basal recording. (2-way ANOVA post hoc LSD test).

**Figure 2:**
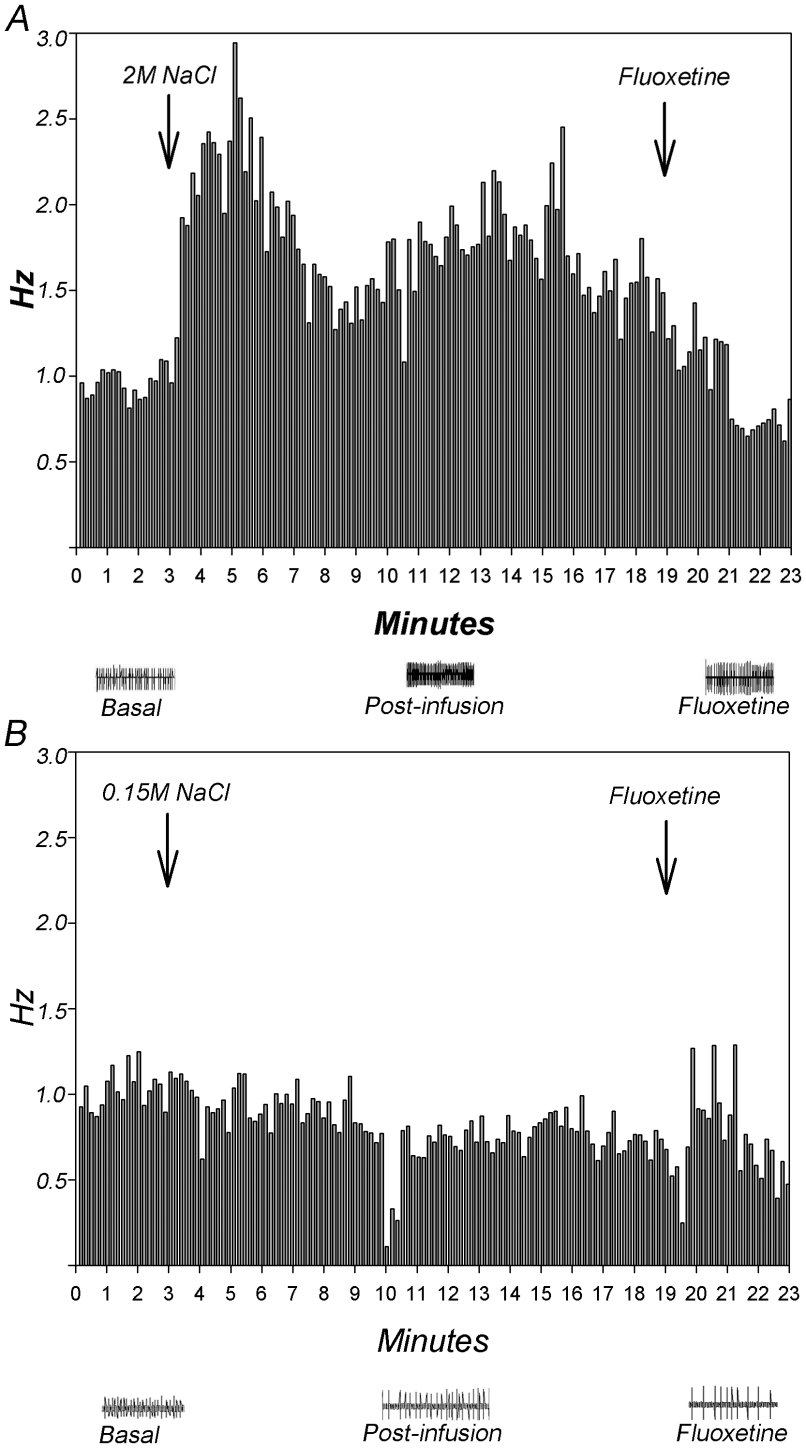
Histograms showing the effect of NaCl sc infusion on electrical activity of putative 5HT-DRN neurons. Fragments recording activity at basal, and after NaCl and fluoxetine infusion are also shown (mV vs ms). A)- 2 M NaCl sc infusion, B)- 0.15 M NaCl sc infusion.

The effect of a 2 M NaCl infusion was tested in 20 putative serotonergic neurons of the DRN. Of the 20 neurons tested with s.c. NaCl 2 M infusion, 14 (70%) displayed an excitatory response, 3 (15%) an inhibitory response and 3 (15%) did not respond. Of the 16 neurons infused with isotonic saline, 3 (19%) displayed an excitatory response, 6 (37.5%) an inhibitory response and 7 (43.5%) did not respond. Taking into account this heterogeneity of responses, the quantitative analysis was done comparing the excitatory putative 5HT neurons of the sodium overload vs control group. The analysis showed a significant interaction between the treatment and time (F_(18,504) = _5.82; P = 0.001). That is, there is a significant differential response in excitatory neuronal firing of the 5HT cells of rats infused with a hypertonic solution of sodium (2 M NaCl) compared to those infused with isotonic saline (0.15 M NaCl) (Figs. 1 and 2). Post hoc analysis indicated that the firing rate of putative serotonergic neurons in rats infused with 2 M NaCl increased significantly 3 and 4 minutes after the infusion started compared to the 0.15 M NaCl injected group and to baseline, respectively. In contrast, the treatment with isotonic saline induced a tendency to decrease the firing rate of serotonergic neurons, being significantly different in the last point of time. Besides, in those neurons that showed an inhibitory response induced by 2 M NaCl, inhibition was observed 4 minutes after the infusion began compared to baseline level.

**Figure 3:**
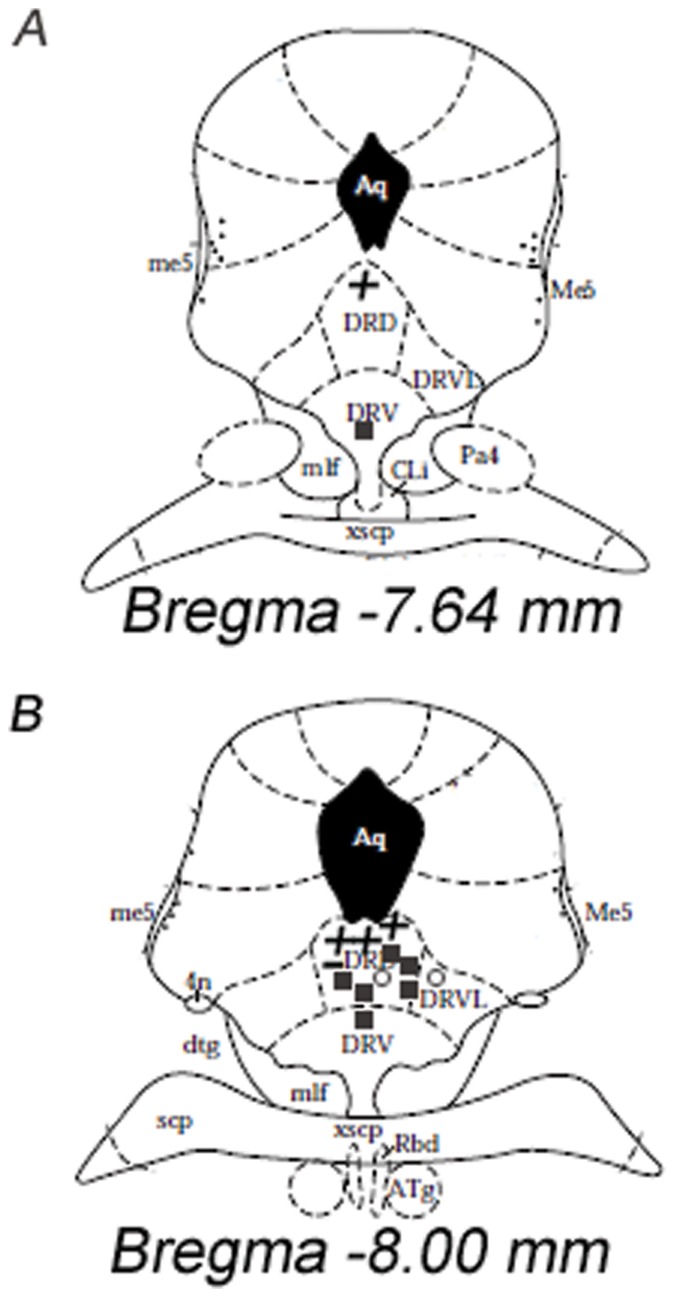
Location of recording sites within the DRN from 2 M NaCl or 0.15 M NaCl infused rats. The recording sites are represented within plates 48 and 50 from Paxinos and Watson (1997) corresponding with −7.64 mm (A) and −8.00 mm (B) distance from bregma, specifying the excitatory (+), inhibitory (−), control () and neutral (Ο) responses. DRD: dorsal subdivision of dorsal raphe nucleus, DRVL: ventrolateral subdivision of dorsal raphe nucleus, DRV: ventral subdivision of dorsal raphe nucleus, Aq: cerebral aqueduct.

Figure 3 shows the representative plots at a rostral (3.A) and caudal level (3.B) of DRN from subjects with sodium or control overload, to illustrate the precise location of the 14 recording sites where the dye injection was successfully delivered, specifying the different responses: excitatory (+), inhibitory(−), control () and neutral (Ο) responses. In the remaining cases we have only an estimation of the recording site location; however all were approximately sited within the dorsal, ventral and ventrolateral subdivisions of the DRN between −7.8 mm and −8.0 mm from bregma.

### Experiment 2: Physiological Characterization of the Response to Sodium Overload

#### Water intake induced by SO

**Figure 4:**
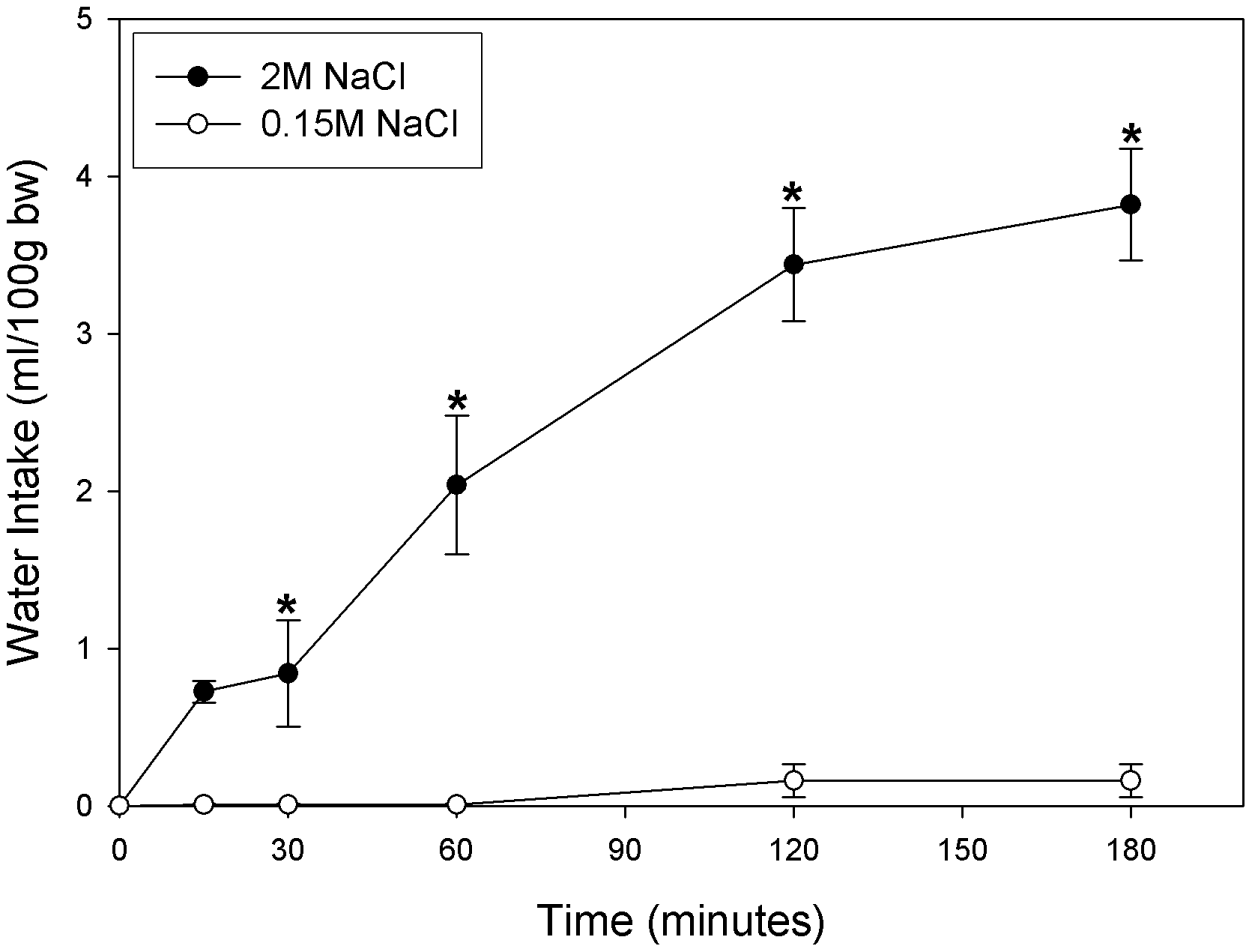
Cumulative volume of water drunk during the intake test (3 h) after 2 M NaCl or 0.15 M infusion. Values are means ± SE. *P<0.05 vs. Control group (n = 9).

As previously described, water intake significantly increased in the 2 M NaCl injected group compared to the 0.15 M NaCl group (Fig. 4). That is, the interaction between treatment factor and time was statistically significant (F_4,64_ = 4.059; p = 0.005), increasing the volume of water drunk by 2 M NaCl rats.

Sodium overload rats started to drink water 31 minutes after 2 M NaCl infusion (means of 9 rats). Most of the control rats did not drink water during the test of 180 minutes (6 rats), and those who drank water started to drink 82 minutes after 0.15 M NaCl injection (means of 3 animals).

### Plasma Protein Concentration. Osmolality and Plasma Sodium and Urinary Concentration

In order to infer whether there are blood volume changes after s.c. saline infusions, plasma protein concentration was examined. No significant differences were observed in plasma protein concentration for treatment factor or time, suggesting that there are no changes in blood volume between groups or baseline levels (Table 1).

**Table 1:** Plasma sodium and protein concentration and plasma osmolality after 2

Time (min)	Plasma Sodium Concentrationmeq/l		Plasma Osmolalitymosmol/KgH_2_O		Plasma Protein Concentrationg/dl		
	NaCl 0.15 M	NaCl 2 M	NaCl 0.15 M	NaCl 2 M	NaCl 0.15 M	NaCl 2 M	
0	146.2±1.0	146.2±1.0	296.91±0.55	296.91±0.55	6.23±0.28	6.23±0. 28	
5	145.1±1.2	148.4±1.1*	287.81±0.89	298.38±0.68*	6.62±0. 34	6.14±0.30	
10	144.5±1.1	151.4±1.0* **^+^**	298.06±0.78	309.35±0.61* **^+^**	6.44±0.32	6.10±0.28	
15	142.7±1.2	148.2±1.1*	296.78±0.89	303.16±0.68* **^+^**	6.18±0.34	6.32±0.30	

Values are means ± SE; n = 5.

* P<0.05 Significantly different between NaCl 2 M and NaCl 0.15 M groups.+P<0.05 Significantly different from baseline levels (time 0).

#### Plasma osmolality and electrolytes

As expected, 2 M NaCl infusions significantly increased plasma sodium concentration and osmolality. The ANOVA for plasma sodium concentration indicated a significant interaction between treatment factor and time (F_3, 64_ = 4.3; p = 0.008). Plasma sodium concentration significantly increased at 5, 10, 15 minutes compared to the control group and also increased at 10 minutes compared to baseline levels (Table 1).

The analysis of plasma osmolality also showed a significant interaction between treatment factor and time (F_3, 64_ = 3.95; p = 0.012). As shown in Table 1, an increase was observed in the sodium overload group at 5, 10, 15 minutes after 2 M NaCl s.c. infusion compared to the 0.15 M NaCl group, and at 10 and 15 minutes compared to baseline levels. The percentage increase of plasma osmolality was 3–4% compared to baseline levels.

#### Renal response induced by sodium overload

Sodium overloaded rats had a different renal response compared to the control group during the 180 minutes after s.c. injection (Table 2). Three of the nine rats injected with isotonic saline did not even urinate, while all the rats infused with hypertonic NaCl showed this response. As shown in Table 2, a significant increase in sodium, chloride and potassium renal excretion was observed after 2 M NaCl s.c. infusion compared to the control infused group. However, no difference was found between groups in urine osmolality.

**Table 2:** Renal response after 2

	Unit	0.15 M NaCl	2 M NaCl
Urinary volume	ml. • 100 g bw^−1^ ·3 h	0.533 (±0.128)	3.105 (±0.255)*
Na^+^ Excretion	meq. • 100 g bw^−1^	0.047 (±0.015)	0.699 (±0.057)*
Cl^−^ Excretion	meq. • 100 g bw^−1^	0.446 (±0.016)	0.798 (±0.071)*
K^+^ Excretion	meq. • 100 g bw^−1^	0.038 (±0.015)	0.143 (±0.024)*
Osmolality	mosmol. • bw^−1^	692.579 (±65.895)	693.826 (±27.407)
		*n = *6	*n = *9

Values are means ± SE; n = 5.

* P<0.05 Significantly different between groups.

### Plasma OT Concentration

**Figure 5:**
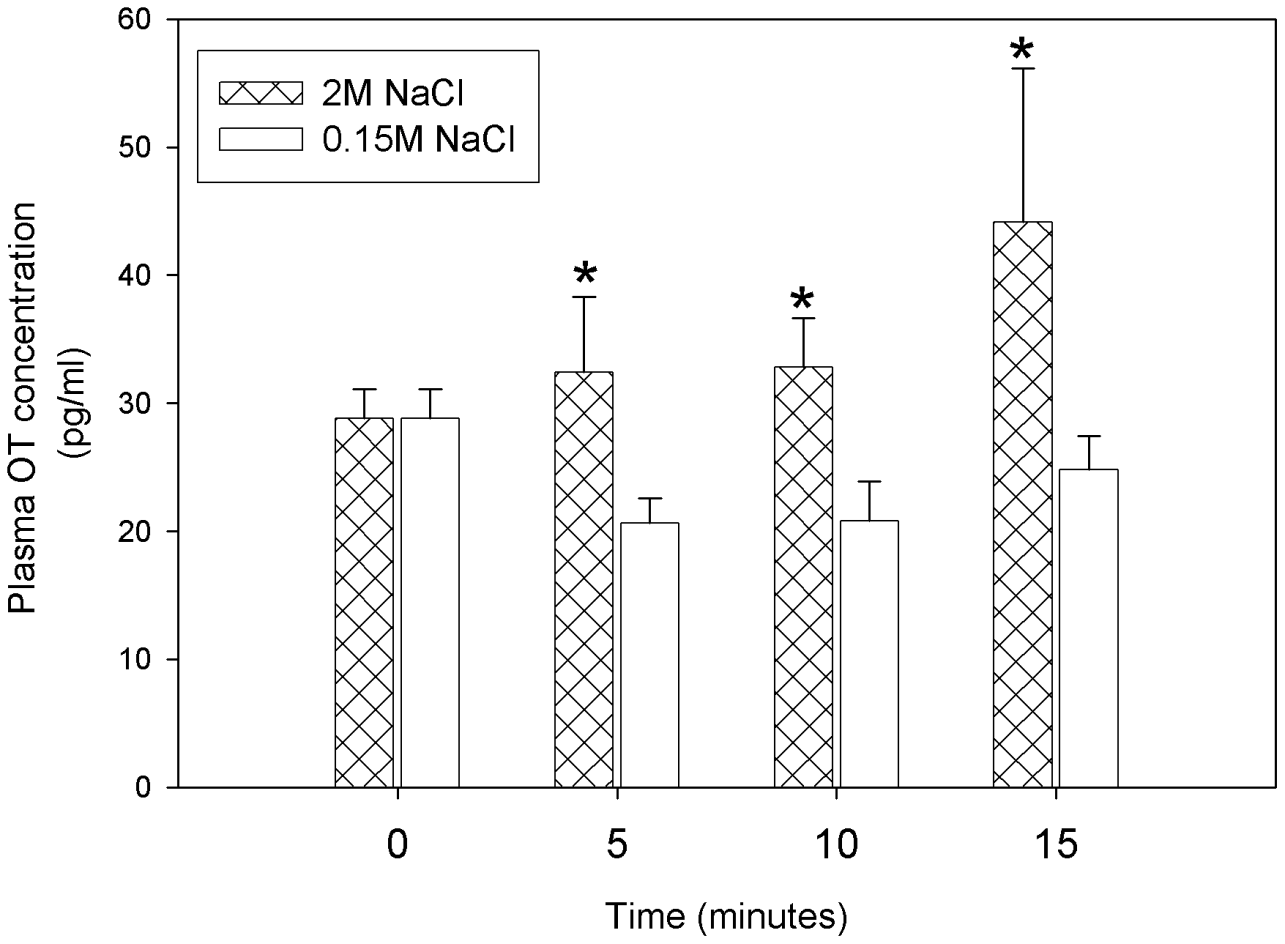
Plasma OT concentration before and 5, 10 and 15 minutes after 2 M NaCl or 0.15 M NaCl infusion. Values are means ± SE. The 2 way ANOVA indicated that the main treatment factor was statistically significant (F1, 45 = 8.3; p = 0.006) *P<0.05 difference from 0.15 M NaCl group.

As previously demonstrated [5], [4], hypertonic sodium infusion changed plasma OT concentration compared to the control group.The ANOVA indicated that the main treatment factor was statistically significant (F_1, 45_ = 8.305; p = 0.006), but the interaction between the treatment factor and time did not reach significant levels (Fig. 5).

### Experiment 3: Brain Pattern of Fos-ir and Double–immunolabeled Cells (Fos-5HT and Fos-OT) after s.c. Infusion of 2 M NaCl vs 0.15 M NaCl Solutions

#### DRN

**Figure 6:**
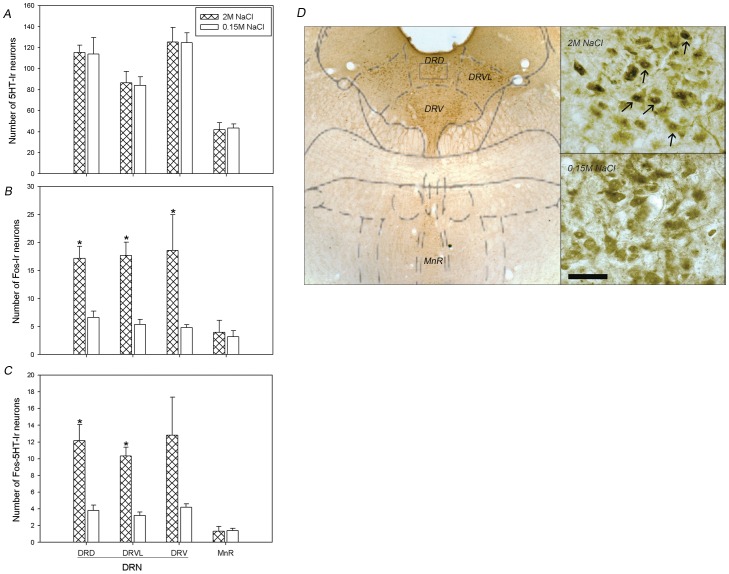
Brain Pattern of Fos-ir and Double–immunolabeled cells, Fos-5HT, after SO. A)- Average number of neurons single-labeled for 5HT, B)- Fos and C)-double-labeled for Fos and 5HT, along the median raphe nucleus and dorsal, ventrolateral and ventral subdivisions of DRN, after s.c. injections of 2 M NaCl or 0.15 M NaCl. Values are means ± SE. *P<0.05 significant differences between sodium overload (2 M NaCl) vs control (0.15 M NaCl) groups. D)- Schematic photomicrographs of DRN and MnR analyzed sections (left panel), illustrating the effects of s.c. injections of 2 M NaCl or 0.15 M NaCl on *c-fos* expression in serotonergic neurons of DRN (upper and bottom right panels, respectively). Small square within DRD indicates the region photographed at higher magnification (40X), and shown in right panels. Fos-5HT immunolabeled cells within the DRD are indicated by arrows (upper right panel). Scale Bar: 50 µm.

As observed in our previous studies, greater activation was found at medial level of the DRN, which includes its dorsal, ventral and ventrolateral subdivisions. As expected based on previous results, sodium overload produced a significant increase in the number of Fos immunoreactive neurons in the DRD, DRV, and DRVL subdivisions of DRN. The number of double-labelled (Fos-5HT) cells is also increased in the DRD and DRVL regions of DRN (Fig. 6). No significant differences were observed in the Fos-ir and Fos-5HT neurons in the other raphe nuclei such as MnR (Figs. 6 B and C respectively). We also analyzed the number of 5HT neurons in both groups; however we did not observe any significant differences in these analyzed areas (Fig. 6A).

#### SON and PVN

**Figure 7:**
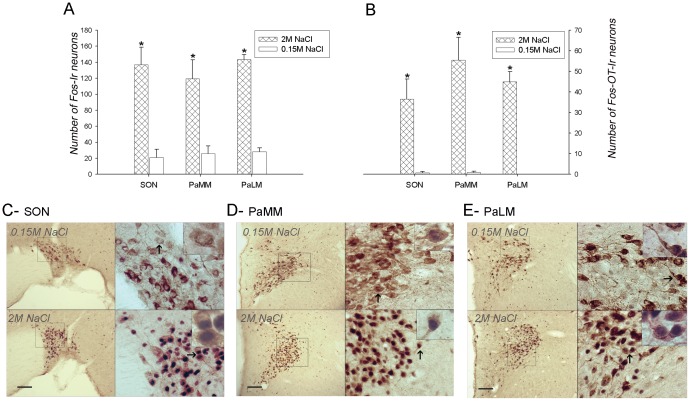
Brain Pattern of Fos-ir and Double–immunolabeled cells, Fos-OT, after SO. A)- Average number of Fos immunoreactive neurons and B)- Fos-OT immunolabeled neurons in the supraoptic nucleus (SON), and paraventricular hypothalamic nucleus along the lateral magnocellular and medial subdivisions (PaLM and PaMM, respectively), after s.c. injections of 2 M NaCl or 0.15 M NaCl. Values are means ± SE. *P<0.05 significant differences between overload (2 M NaCl) vs. control (0.15 M NaCl) groups. The bottom panels photomicrographs (C,D,E) are showing the pattern of double Fos-OT immunoreactive cells in the SON, C)-, and paraventricular hypothalamic nucleus along the medial (PaMM, D) and lateral magnocellular (PaLM, E) subdivisions in control (upper sections) and sodium overload animals (bottom sections). The left panels illustrate the distribution of these immunoreactive cells at low magnification (10x). Small squares in these panels indicate regions photographed at higher magnification (40x, right panels), and indicated by arrows are the cells photographed at higher magnification (100×). Scale Bar: 100 µm.

Fos expression in the SON was significantly increased in sodium overloaded animals compared to the control group. As previously shown [5], [4], the number of double-immunolabeled neurons for Fos and oxytocin was also significantly increased in the 2 M NaCl group (Fig. 7).

Within the medial (PaMM) and lateral (PaLM) magnocellular subdivisions of the PVN, a significant increase in Fos and Fos-OT immunoreactive cells was found, 90 min after s.c. 2 M NaCl infusion (Fig. 7).

### Brain Pattern of Fos-ir Neurons in Other Nuclei Involved in Sodium Balance Regulation

**Figure 8:**
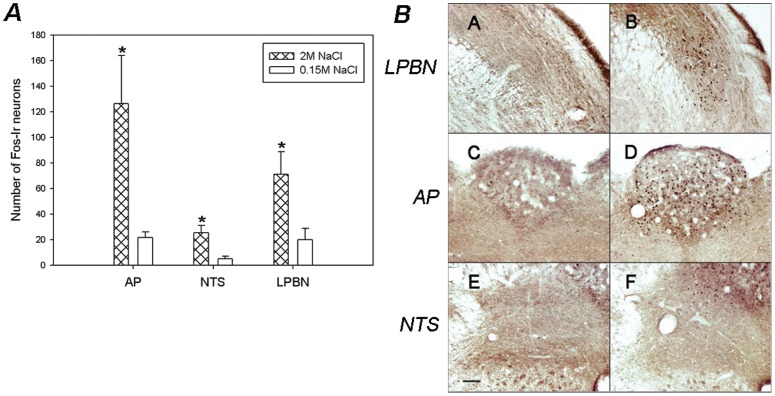
Brain Pattern of Fos-ir in the brainstem nuclei after SO. A)- Average number of Fos-immunoreactive neurons in the NTS, AP and LPBN after s.c. injections of 2 M NaCl or 0.15 M NaCl. Values are means ± SE. *P<0.05 significantly different from 0.15 M NaCl group. B)- Photomicrographs showing the pattern of Fos-immunoreactivity within the LPBN (A–B), AP (C–D) and NTS (E–F), after s.c. injections of 2 M NaCl (B,D,F) or 0.15 M NaCl (A, C, E). Scale Bar: 100 µm.

*Brainstem:* The *t-test* analysis indicated that sodium overload produces a significant increase in the number of Fos immunoreactive cells along the AP, NTS and LPBN compared to control animals (Fig. 8).

#### Lamina terminalis

**Figure 9:**
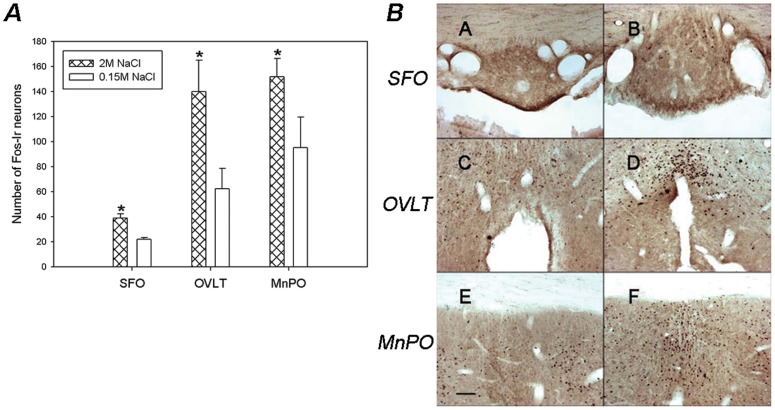
Brain Pattern of Fos-ir in the Lamina Terminalis nuclei after SO. A)- Average number of Fos-immunoreactive neurons in the SFO, OVLT and MnPO after s.c. injections of 2 M NaCl or 0.15 M NaCl. Values are means ± SE. *P<0.05 significantly different from 0.15 M NaCl group. B)- Photomicrographs showing the pattern of Fos-immunoreactivity within the SFO (A–B), OVLT (C–D) and MnPO (E–F), after s.c. injections of 2 M NaCl (B,D,F) or 0.15 M NaCl (A, C, E). Scale Bar: 100 µm.

The 2 M NaCl group showed a significant increase in the number of Fos-ir neurons along the circumventricular organs of the lamina terminalis, OVLT and SFO, compared to the control group. A significant increase in the number of Fos-ir cells of experimental group was also observed in the ventral part of the MnPO (Fig. 9).

#### Central Extended Amygdala (ExA)

**Figure 10:**
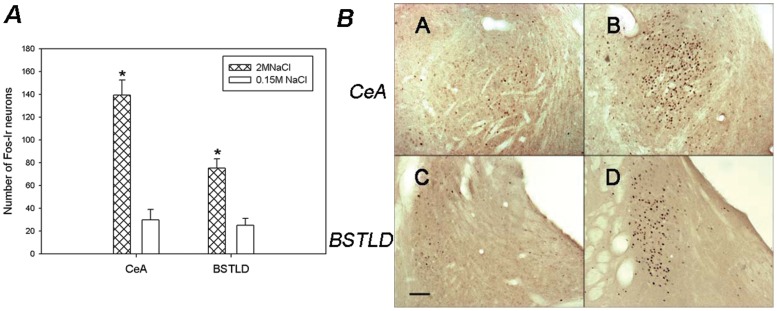
Brain Pattern of Fos-ir in the Central Extended Amygdala nuclei after SO. A)- Average number of Fos-ir neurons in the CeA and BSTLD after s.c. injections of 2 M NaCl or 0.15 M NaCl. Values are means ± SE. *P<0.05 significantly different from 0.15 M NaCl group. B)- Photomicrographs showing the pattern of Fos-immunoreactivity within the CeA (A–B) and BSTLD (C–D), after s.c. injections of 2 M NaCl (B,D,F) or 0.15 M NaCl (A, C, E). Scale Bar: 100 µm.

A significantly increased number of activated neurons were observed within the central extended amygdala nuclei, CeA and BSTLD of sodium-overloaded rats, in comparison with control rats (Fig. 10).

### Brain Pattern of Fos-ir Neurons in other Nuclei not Involved in Sodium Balance Regulation

In order to demonstrate that the sodium-overload induced effect is selective to brain regions of interest, and not a global elevation in neuronal activity throughout the brain we included the analysis of others nuclei such as AD and AcbC. In both areas we did not observed significantly differences between sodium overload and control groups (AD: mean of 2 M NaCl: 15.17, mean of 0.15 M NaCl: 15.4, p = 0.97 and AcbC: mean of 2 M NaCl: 20.8, mean of 0.15 M NaCl: 22, p = 0.91).

## Discussion

The results of this study allow us to determine in an “*in vivo*" model that DRN 5HT neurons increase their firing frequency during an increase in systemic sodium concentration and osmolality, matching the pattern of Fos-5HT double immunostaining. Therefore, both electrical activity (analyzed by *in vivo* extracellular recording) and c-fos expression within serotonergic cells of the DRN increased after a hypernatremic/hyperosmolar state.

The endocrine, renal and behavioral responses were also analyzed in the same model and the brain areas and the oxytocinergic PVN-SON groups of neurons activated after a sodium overload were identified (by single and double immunolabeling).

Our data provide new evidence regarding the activation of specific groups of serotonergic cells within the DRN during increases of plasma sodium concentration and osmolarity not associated to volume expansion.

The electrophysiological evidence supports previous immunohistochemical and pharmacological results that involved serotonergic pathways modulating sodium intake and renal excretion after different hydroelectrolytic disturbances. Our previous reports demonstrated serotonergic system involvement in the inhibitory control of sodium appetite induced by peritoneal dialysis (PD). Fos-ir decreased in serotonergic cells 24 hs after PD (during the appetitive phase) compared to non-depleted animals, while Fos-ir increased during the satiety phase when animals were in process of reestablishing body sodium status by ingesting sodium salts [9], [11], [12]. According to these, it has been shown that fos expression depends on the temporal features of action potential patterns. For example in cultured dorsal root ganglion cells, immediate-early gene activation was inversely correlated with the burst –intervals of action potentials [30]. Our previous results also demonstrate that the 5HT system is involved in the regulation of renal responses. Serotonergic receptor blockade with LPBN injections of methysergide reduced the increase in urinary volume and sodium and potassium excretion induced by EVE, while injections of the serotonergic 5HT2A and 5HT2C receptor agonist, 2.5-dimethoxy-4-iodoamphetamine hydrobromide, enhanced the effects of BVE on Na_ and K_ excretion and urinary volume [20]. Decreased levels of 5HT and its metabolite 5-hydroxyindoleacetic acid were also observed within the raphe nucleus 15 min after EVE [14]. Finally, the activation and the increase in serotonergic DRN firing frequency after a body sodium overload may be interpreted as these cells participate in the behavioral osmoregulatory response [9]–[11]. It would also influence renal and endocrine responses, increasing plasma OT and atrial natriuretic peptide and consequently increasing urine output and sodium and potassium renal excretion, as often reported after an EVE or after induced sodium intake [10], [11], [20].

These data together suggest that the serotonergic system at DRN level is modulated by body sodium status and is therefore participating in its regulation. In sum, the increased activity and firing frequency of putative serotonergic cells of the DRN after body sodium overload may reflect how this system mediates the behavioral, renal and endocrine responses for reestablishing body sodium balance.

Neuroanatomical evidence indicates that the DRN sends 5HT projections to the LPBN [14], [31], forming a key pathway to regulate homeostatic responses under hydroelectrolyte balance alterations. Previous investigations also demonstrated that serotonergic mechanisms in the LPBN play an inhibitory role in controlling sodium appetite following a variety of dipsogenic and/or natriorexigenic stimuli [17], [32]–[35]. Both the LPBN and the DRN receive afferents from the LT nuclei and their cells are activated not only after SO but also during different body sodium balance changes [13], [12]. That is, hypertonicity would be detected by the LT, which sends projections directly to the DRN and LPBN, increasing the activity of DRN 5HT cells, which also send efferents to the LPBN, modulating its activity and consequently sodium appetite.

As expected, SO significantly increased plasma sodium concentration and osmolality. The highest increase was observed at 10 minutes of NaCl injection. However, no differences in plasma protein concentration were observed, suggesting that this protocol of SO does not involve an EVE.

As previously described [36]–[39], an increase of 1–2% in plasma osmolality produces the activation of a central circuit that induces thirst in order to reestablish normal osmolality. Similarly, we observed that the SO group consumed three times more water than the control group during the 3 h drinking test. Renal response is also implicated in plasma tonicity regulation. In agreement with previous reports, we found that SO enhanced volume and electrolyte excretion [39], [40]. High sodium excretion is in part a consequence of the natriuretic effect of plasma oxytocin, which was also found to be elevated. These data confirm previous studies which showed that OT is released after hyperosmotic stimulus [41]–[43].

The observed variations in osmolarity and plasma sodium concentration produced by subcutaneous sodium overload are detected by highly specialized neurons, able to translate these changes to electrical signals which activate CNS areas involved in the control of water and salt intake and excretion [44]–[46]. There is a general agreement that osmosensory transduction is primarily mediated by cells of the two circumventricular organs located in the lamina terminalis: the SFO and the OVLT [44], [45], [47]–[50]. These areas send information to the DRN [13] and hypothalamic nuclei such as PVN, SON and MnPO [50]–[52]. Then they may activate the magnocellular neurons of the PVN and SON, increasing the plasma concentration of OT. As previously described in the results section, sodium overload also increased Fos-ir and Fos-OT positive cells in the SON after 2 M NaCl infusion; however, isotonic infusion did not produce any change in oxytocinergic cell activity.

Different areas of the brainstem such as the NTS, AP, LPBN, which mediate peripheral satiety and osmoregulatory signals to modulate fluid intake and neurohypophyseal hormone secretion, showed increased Fos immunoreactivity after SO, and structures of the extended amygdala complex, such as the CeA and bed nucleus of the stria terminalis (BST), which are involved in the processing of integrated signals related to sodium appetite behavior, are activated by SO [4]–[8], [53], [54]. These data give new evidence for BST involvement during systemic hypertonicity states associated with normo-volemia. Previous studies indicated that both the CeA and BST sub-nuclei are also activated by isotonic and hypertonic EVE and are involved in thirst and sodium appetite control [10], [55]–[57].

In conclusion, taking into account these results and other studies, we can speculate on the possible brain circuit involved in regulatory responses during a SO without volume expansion. Lamina terminalis structures detect humoral changes produced by SO such as plasma hormones, Na concentration and osmolality changes. The circumventricular organs of the LT send projections to the hypothalamus (SON and PVN), brainstem nuclei (LPBN, NTS, AP and DRN) and central extended amygdala complex. LT projections, among others, activate the oxytocinergic neurons of SON and PVN, increasing plasma OT concentration in order to promote sodium excretion. SO produces an increase in Fos-ir and electrical activity of 5HT DRN neurons. These cells may control body sodium status, inhibiting sodium appetite and increasing natriuresis.
