# Correction: Body Sodium Overload Modulates the Firing Rate and Fos Immunoreactivity of Serotonergic Cells of Dorsal Raphe Nucleus

**DOI:** 10.1371/annotation/1f6a85aa-dcbb-40f6-910b-a1189cefe56b

**Published:** 2014-01-03

**Authors:** Andrea Godino, Soledad Pitra, Hugo F. Carrer, Laura Vivas

There is an error in the author contributions, please note that the last sentence of the author contributions section should read: Technical assistance for electrophysiology experiments: HFC. 

Additionally, an error was introduced into the legend of Figure 3 during the preparation of this article for publication. The legend that represents the control responses is missing. The correct first sentence of the legend is: The recording sites are represented within plates 48 and 50 from Paxinos and Watson (1997) corresponding with −7.64 mm (A) and −8.00 mm (B) distance from bregma, specifying the excitatory (+), inhibitory (−), control (∎) and neutral (Ο) responses.

